# Chiroptical Activities of Low‐Dimensional Lead‐Free Chiral Halide Perovskites with White‐Light Emission

**DOI:** 10.1002/smsc.202500034

**Published:** 2025-05-15

**Authors:** Min‐Han Tsai, Chia‐Hsiang Chuang, Pei‐Hsuan Lo, Wei‐Yun Zeng, Chun‐Yao Huang, Lan‐Sheng Yang, Yu‐Chiang Chao

**Affiliations:** ^1^ Department of Physics National Taiwan Normal University Taipei 116 Taiwan

**Keywords:** chiral halide perovskites, circular dichroisms, circularly polarized luminescences, lead free, low dimensions

## Abstract

Chiral halide perovskites have attracted considerable attention due to their intrinsic chirality‐induced circular dichroism (CD), circularly polarized luminescence (CPL), and spin selectivity. However, chiroptical activities of low‐dimensional lead‐free chiral halide perovskites are hardly observed, especially for those with white‐light emission. Herein, lead‐free halide perovskites with different ratios of 0D Cs_3_Cu_2_I_5_ and 1D CsCu_2_I_3_ are realized. Chiroptical activities are introduced into these films by post‐treatment with r‐/s‐methylbenzylammonium iodide to realize lead‐free chiral halide perovskites. The absorption, photoluminescence, photoluminescence excitation, CD, and CPL spectra of these films are investigated. The Commission Internationale de L’Eclairage chromaticity coordinates (0.33, 0.33) are obtained when the excitation wavelength is 300 nm. Large Stokes shifts and broadband emission are observed and attributed to the presence of self‐trapped excitons. At room temperature, intrinsic chirality‐induced CD signals are observed without the application of an external magnetic field, indicating the presence of chirality in the low‐dimensional lead‐free chiral halide perovskites. Room‐temperature CPL is also observed from the low‐dimensional lead‐free chiral halide perovskites, but only from the Cs_3_Cu_2_I_5_ component. This is attributed to the fact that CsCu_2_I_3_ does not produce CPL and spin‐polarized excitons do not transfer from Cs_3_Cu_2_I_5_ to CsCu_2_I_3_.

## Graphical abstract

Lead‐free chiral halide perovskite films composed of Cs_3_Cu_2_I_5_ and CsCu_2_I_3_ are prepared. White emission with Commission Internationale de L’Eclairage chromaticity coordinates (0.33, 0.33) is obtained. Circular dichroism and circularly polarized luminescence are observed at room temperature.

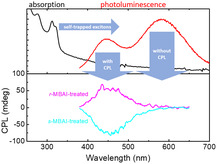

## 1 Introduction

Halide perovskites have attracted considerable attention for their potential applications in solar cells, light‐emitting diodes (LEDs), and photodetectors. In addition to red, green, and blue emission, LEDs with white light emission are an important focus for lighting and backlighting applications.^[^ ^1^, ^2^, ^3^, ^4^ ^]^ Many materials and methods have been developed to produce white light emission from a single‐emissive layer.^[^ ^5^, ^6^, ^7^, ^8^, ^9^, ^10^, ^11^, ^12^, ^13^ ^]^ Among these materials, a mixture of 0D Cs_3_Cu_2_I_5_ and 1D CsCu_2_I_3_ is attractive because of its lead‐free nature, ease of fabrication, broadband emission, and abundance on Earth.^[^ ^11^, ^12^, ^13^, ^14^ ^]^

Chirality, also known as mirror asymmetry, is a geometrical property of an object whose mirror image is not identical to itself.^[^ ^15^ ^]^ Chirality is one of the important fundamental properties of many biomolecules and their assemblies, such as proteins and deoxyribonucleic acid.^[^ ^16^ ^]^ Organic, inorganic, and organic–inorganic hybrid materials also exhibit apparent chiral properties.^[^ ^17^, ^18^ ^]^ Recently, chirality has also been observed in organic–inorganic halide perovskites containing chiral molecules.^[^ ^19^, ^20^, ^21^, ^22^ ^]^ Interesting properties such as circular dichroism (CD), circularly polarized luminescence (CPL), chiral‐induced spin selectivity effect, and chiro‐spintronics have been demonstrated in chiral halide perovskites.^[^ ^23^, ^24^, ^25^, ^26^ ^]^ Chiral perovskites exhibiting CPL have attracted considerable interest for their potential applications in 3D displays and quantum‐based optical computing.^[^ ^27^, ^28^ ^]^

Chiral halide perovskites with both white‐light emission and CPL are still rare. Chiral 1D (C_5_H_14_N_2_)PbCl_4_·H_2_O, prepared from Pb(AC)_2_·3H_2_O and *R*‐3‐aminopiperidine dihydrochloride in dilute aqueous HCl solution, exhibited white‐light emission with Commission Internationale de L’Eclairage (CIE) chromaticity coordinates of (0.39, 0.37).^[^ ^29^ ^]^ Second‐harmonic generation signals and CD spectra have been reported.

However, no CPL information was reported. Chiral 2D (*R*‐(+)‐β‐MPEA)_2_PbBr_4_ and (*S*‐(−)‐β‐MPEA)_2_PbBr_4_ (β‐MPEA = beta‐methylphenethylamine) exhibited white‐light emission with CIE chromaticity coordinates of (0.31, 0.36) and (0.33, 0.37), respectively.^[^ ^10^ ^]^ CD spectra and time‐resolved photoluminescence (PL) were reported, but no CPL signals were reported. Recently, eight hybrid lead(II) bromide perovskitoids with CIE chromaticity coordinates close to the standard white‐light were prepared.^[^ ^30^ ^]^ CPL spectra were recorded and the dissymmetric factor (*g*_lum_) of 8 × 10^−3^ was obtained. Chiral 1D (*R*‐C_5_H_14_N_2_)PbBr_4_·H_2_O and (*S*‐C_5_H_14_N_2_)PbBr_4_·H_2_O showed yellowish‐white CPL emission with a *g*_lum_ value of 1.8 × 10^−3^.^[^ ^31^ ^]^ The yellowish‐white light PL spectra with CIE chromaticity coordinates of (0.429, 0.452) and (0.427, 0.449) were observed. However, these results are all based on chiral halide perovskites containing toxic lead. Lead‐free chiral halide perovskites with chiroptical activity are still rare.

In this work, chirality was incorporated into the low‐dimensional lead‐free chiral halide perovskite films, composing of 0D Cs_3_Cu_2_I_5_ and 1D CsCu_2_I_3_. The chiroptical activities of these chiral halide perovskite films were investigated. Precursor solutions of cesium iodide (CsI) and copper(I) iodide (CuI) with different molar ratios were used to prepare the halide perovskite films. The absorption, PL, and PL excitation (PLE) spectra of these films were studied to understand the influence of the molar ratio of CsI to CuI in the precursor solutions on the composition and optical properties of the thin films composed of Cs_3_Cu_2_I_5_ and CsCu_2_I_3_. The CIE chromaticity coordinates of (0.33, 0.33) were obtained when the excitation wavelength was 300 nm. Large Stokes shifts and broadband emission were observed and attributed to the presence of self‐trapped excitons (STEs). To introduce the chirality to the halide perovskite films, the films were then post‐treated with *r*‐/*s*‐methylbenzylammonium iodide (*r*‐/*s*‐MBAI). CD and CPL spectra of this low‐dimensional lead‐free chiral halide perovskite were observed at room temperature. Symmetric CD signals were observed in the absence of an external magnetic field, representing the intrinsic chirality‐induced CD features. The CPL spectra were also observed in the same PL wavelength range, indicating that the spin‐polarized free excitons can transfer to the STE state without losing their spin information through an energy and spin funneling process. However, the CPL signal only comes from the Cs_3_Cu_2_I_5_ component. This is attributed to the fact that CsCu_2_I_3_ does not produce CPL and spin‐polarized excitons do not transfer from Cs_3_Cu_2_I_5_ to CsCu_2_I_3_.

## 2 Results

### 2.1 Composition and Optical Properties

The lead‐free halide perovskite films were prepared from perovskite precursor solutions containing CsI and CuI in a mixture of dimethylformamide (DMF) and dimethyl sulfoxide (DMSO) (1:1 v/v) or inγ‐butyrolactone (γ‐GBL). Three precursor solutions with CsI to CuI molar ratios of 2.75:2, 1.15:2, and 1.05:2 were prepared for spin coating. (Figure S1, Supporting Information) Toluene was used as an antisolvent during the spin coating process of the precursor solution.

**Figure 1:**
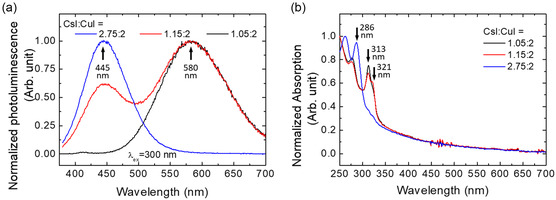
a) PL and b) absorption spectra of perovskite films prepared from precursor solutions with different molar ratios of CsI to CuI.

The PL and absorption spectra of these perovskite films are shown in **Figure** **1**. When the molar ratio of CsI to CuI is changed from 2.75:2 to 1.05:2, the wavelength of the PL peak is redshifted from 445 to 580 nm. For the perovskite film prepared from the precursor with a CsI to CuI molar ratio of 1.15:2, two distinct PL peaks were observed at both 445 and 580 nm. Previous reports have shown that the PL peak at 445 nm is from Cs_3_Cu_2_I_5_, while the PL peak at 580 nm is from CsCu_2_I_3_.^[^ ^11^, ^13^, ^14^, ^32^, ^33^, ^34^, ^35^, ^36^ ^]^ Therefore, the perovskite films prepared from the precursor solutions with CsI to CuI molar ratios of 2.75:2 and 1.05:2 are Cs_3_Cu_2_I_5_ rich and CsCu_2_I_3_ rich, respectively. In addition, previous reports have shown that the absorption peak can be observed at ≈290 and ≈315 nm for Cs_3_Cu_2_I_5_ and CsCu_2_I_3_, respectively.^[^ ^12^, ^14^, ^33^, ^34^, ^36^, ^37^ ^]^ The perovskite film prepared from the precursor with CsI to CuI molar ratio of 2.75:2 and 1.05:2 showed a clear absorption peak at 286 and 313 nm, respectively, confirming their Cs_3_Cu_2_I_5_‐rich and CsCu_2_I_3_‐rich nature. An absorption shoulder at 321 nm was also observed for the perovskite films prepared from the precursor solutions with CsI to CuI molar ratio of 1.15:2 and 1.05:2. The solvent used to dissolve CsI and CuI does not cause any noticeable difference in the PL and absorption spectra. (Figure S2, Supporting Information) All these results clearly indicate that the ratio of Cs_3_Cu_2_I_5_ and CsCu_2_I_3_ in the perovskite film can be easily tuned by the molar ratio of CsI and CuI in the precursor solution. The perovskite film prepared from the precursor with a molar ratio of CsI to CuI of 1.15:2 is a composite of Cs_3_Cu_2_I_5_ and CsCu_2_I_3_. The scanning electron microscopy image shows that the film is a discrete film with grains around 100 nm in diameter (Figure S3a, Supporting Information).

X‐ray diffraction (XRD) patterns were recorded to confirm the above‐mentioned observation, as shown in Figure S4, S5, and S6, Supporting Information. Compared with the XRD patterns shown in the previous report,^[^ ^12^ ^]^ PDF#77‐0069 (CsCu_2_I_3_) and PDF#79‐0333 (Cs_3_Cu_2_I_5_), the perovskite film indeed changes from a Cs_3_Cu_2_I_5_‐rich nature to a CsCu_2_I_3_‐rich nature when the precursor with a molar ratio of CsI to CuI changes from 2.75:2 to 1.05:2.

**Figure 2:**
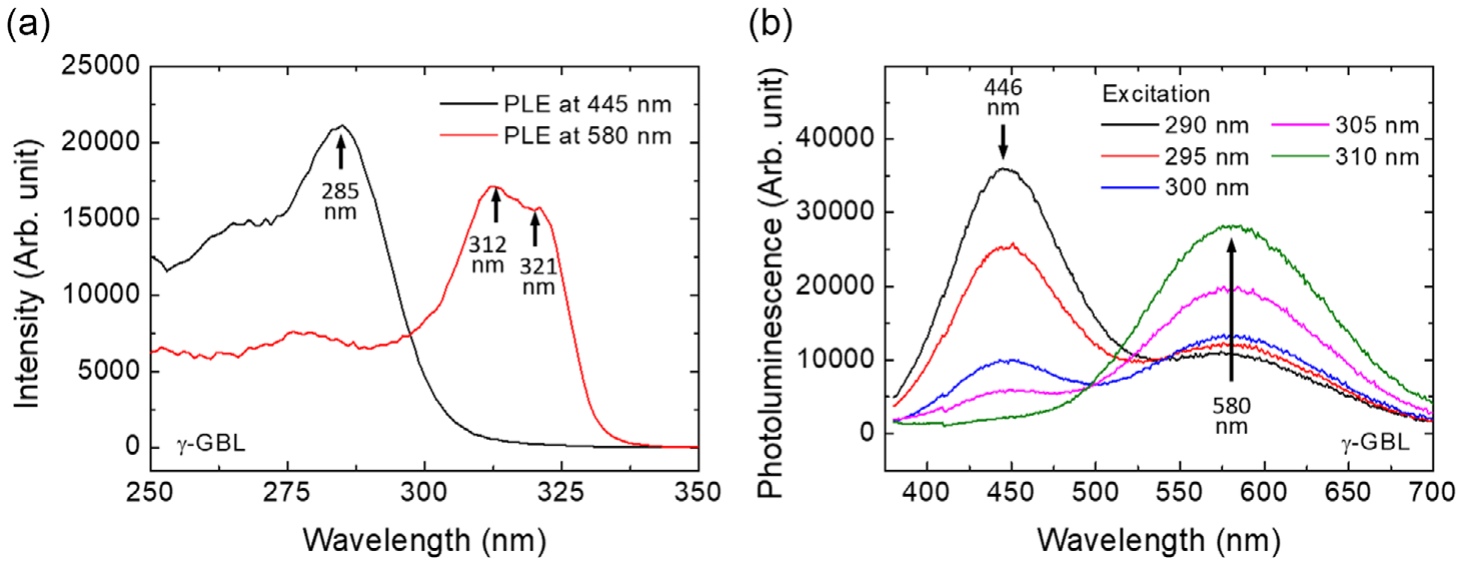
a) PLE spectra measured at 445 and 580 nm. b) PL spectra measured at different excitation wavelengths. The solvent used to prepare the precursor solution isγ‐GBL.

To further confirm the origin of the two PL peaks, PLE spectra were measured at wavelengths of 445 and 580 nm, as shown in **Figure** **2**a and Figure S8a, Supporting Information. The PLE spectrum measured at 445 nm showed a peak at 286 nm, which is similar to the absorption spectrum of Cs_3_Cu_2_I_5_. The PLE spectrum measured at 580 nm showed a peak at 312 nm and a shoulder at 321 nm, which is similar to the absorption spectrum of CsCu_2_I_3_. These results are consistent with previous reports and indicate the coexistence of Cs_3_Cu_2_I_5_ and CsCu2I_3_ in the perovskite film prepared from the precursor with a CsI to CuI molar ratio of 1.15:2.^[^ ^13^, ^14^, ^34^, ^37^ ^]^ Due to the difference in the PLE spectra of Cs_3_Cu_2_I_5_ and CsCu_2_I_3_, the PL spectra and the CIE chromaticity coordinates change with the excitation wavelength, as shown in Figure 2b, Figure S7, S8b, and S9, Supporting Information. The CIE chromaticity coordinates change from (0.22, 0.18) to (0.44, 0.47) when the excitation wavelength changes from 290 to 310 nm. When the excitation wavelength is 300 nm, the CIE chromaticity coordinates are (0.33, 0.33), representing the ideal pure white emission. The combination of yellow emission from CsCu_2_I_3_ and blue emission from Cs_3_Cu_2_I_5_ is promising for the production of white emission. All these results indicate that white emission can be achieved by controlling both the molar ratio of CuI to CsI in the precursor solution and the excitation wavelength.

Large Stokes shifts of 159 and 267 nm were observed for the perovskite film prepared from the precursor with CsI to CuI molar ratios of 2.75:2 and 1.05:2, respectively. Broadband luminescence from these films was observed above 400 nm with no noticeable emission near the absorption band edge, suggesting an almost complete energy transfer to the emission state. Previous reports have shown that these apparent Stokes shifts and broadband emission originate from STEs as a result of strong exciton–phonon coupling, band structure, and Jahn–Teller distortion.^[^ ^12^, ^32^, ^34^, ^35^, ^36^, ^37^ ^]^

### 2.2 Chiroptical Properties

To realize lead‐free chiral halide perovskites, we try to anchor chiral molecules on the surface of the perovskite film prepared from the precursor with CsI to CuI molar ratios of 1.15:2. The post‐treatment method was used to introduce chiral molecules onto the lead‐free halide perovskite films. The chirality of the post‐treated perovskite films was measured using a CD spectrometer.

The CD spectra, that is, the difference in absorption of left‐ and right‐circularly polarized (LCP and RCP) light, the absorption spectra, and the absorption dissymmetry factor $g_{abs}=\frac{\Delta A}{A}=\frac{CD\left(m\deg\right)}{32980\times A}$ were recorded.

**Figure 3:**
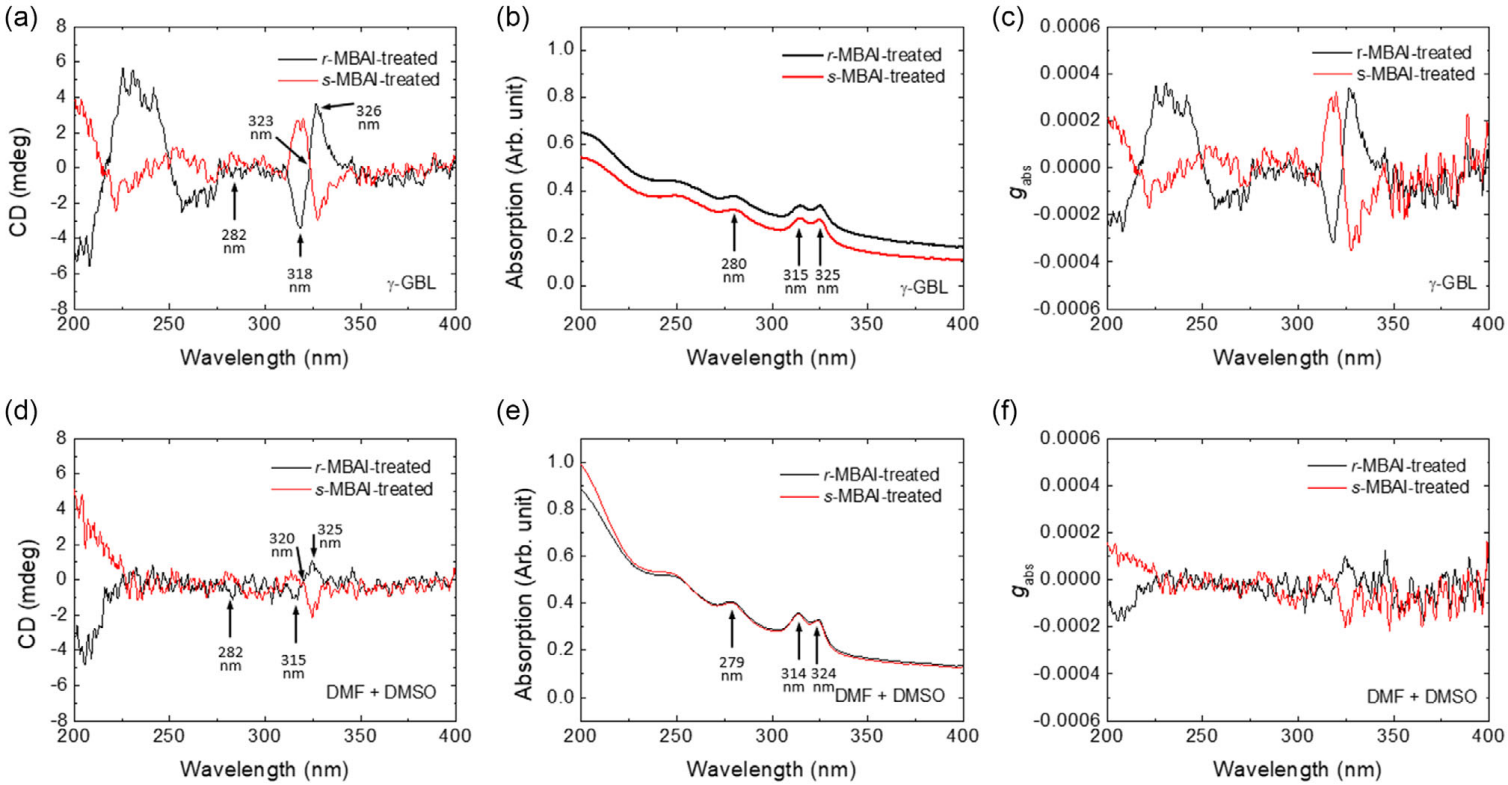
a,d) CD spectra, b,e) absorption spectra, and c,f) *g*_abs_ values of the post‐treated halide perovskite films prepared from the precursor solution in (a),(b),(c)γ‐GBL and (d),(e),(f) DMF + DMSO.

The total absorption of the unpolarized light is represented by *A*. *r*‐methylbenzylammonium iodide (*r*‐MBAI) was first dissolved in toluene, chlorobenzene, chloroform, xylene, and diethyl ether at a concentration of 1.5 mg ml^−1^. The solution containing *r*‐MBA was then applied to the top surface of the perovskite film and left to stand for a period of time before spin drying. As shown in Figure S10, Supporting Information, only the perovskite film post‐treated with *r*‐MBAI toluene solution retained its absorption spectrum and showed a CD signal at the absorption band edge. When the treatment time is longer than 0.5 min, the absorption spectrum changed and the CD signal at the absorption band edge disappeared, as shown in Figure S11, Supporting Information. All these results indicate that both the choice of post‐treatment solvent and the treatment time are important for anchoring the chiral molecule. As a result, using these optimized post‐treatment experimental conditions for *r*‐MBAI and *s*‐MBAI, a clear CD signal can be obtained from both lead‐free halide perovskite films prepared from different precursor solvents, as shown in **Figure** **3**. Mirror‐like features, which is known as the Cotton effect, were observed at the absorption band edge of Cs_3_Cu_2_I_5_ and CsCu_2_I_3_ for the perovskite films post‐treated with *r*‐MBAI and *s*‐MBAI. However, the bisignate CD signal is clearer and *g*_abs_ is larger for the perovskite film prepared fromγ‐GBL. The CD signal shown in Figure 3 differs from the CD spectra of *r*‐/*s*‐MBA and *r*‐/*s*‐MBAI,^[^ ^38^, ^39^, ^40^, ^41^ ^]^ indicating that the optical chirality of *r*‐/*s*‐MBAI has been successfully transferred to the lead‐free halide perovskite films. As shown in Figure S12, Supporting Information, the characteristics of the absorption and PL spectra of the post‐treated perovskite films measured with a spectrofluorometer were approximately the same as those of the pristine perovskite films shown in Figure 1 and S2, Supporting Information. However, the difference in absorption spectra can still be observed (Figure S12b, Supporting Information). From the XRD patterns recorded before and after *r*‐/*s*‐MBAI treatment (Figure S5, Supporting Information), the difference in the intensity of the peaks from 0D Cs_3_Cu_2_I_5_ and 1D CsCu_2_I_3_ increases after r‐/s‐MBAI treatment, which may come from the variations in the 0D Cs_3_Cu_2_I_5_ and 1D CsCu_2_I_3_ composite percentages. Besides, we also found that the PL intensity increased for a large extend, indicating excellent passivation by both enantiomers. The difference in the extent of PL enhancement may also come from the variations in the 0D Cs_3_Cu_2_I_5_ and 1D CsCu_2_I_3_ composite percentages.

Absorption of LCP or RCP light occurs when the scalar product of the electric transition dipole moment (***μ***) and the magnetic transition dipole moment (***m***) has a nonvanishing value.^[^ ^23^ ^]^ The integral of the CD spectrum is proportional to the rotational strength R=Im(μ⋅m). *R* has an opposite sign for *s*‐ or *r*‐configuration in chiral molecules, which have mirror‐image arrangements. Besides, the band‐edge electronic structure and optical selection rule of halide perovskites are usually described by $\left|J,m_{J}\right\rangle$ states of $\left|\frac{1}{2},+\frac{1}{2}\right\rangle$ and $\left|\frac{1}{2},-\frac{1}{2}\right\rangle$, where *J* is the total angular momentum quantum number and *m*_J_ is the magnetic quantum number.^[^ ^42^, ^43^ ^]^ When LCP (σ+) and RCP (σ−) light‐carrying angular momentum of +1 and −1 is absorbed, the absorption process is governed by a selection rule of Δ*m*_J_ = ±1 by conservation of angular momentum. This process allows the selective generation of spin‐polarized excitons with angular momentum projection *J*_z_ = ±1. After the introduction of the chiral organic cation *r*‐/*s*‐MBA, the degeneracy of energy states is broken, leading to the energy‐level splitting and the $\left|\frac{1}{2},+\frac{1}{2}\right\rangle$ state is no longer similar to the $\left|\frac{1}{2},-\frac{1}{2}\right\rangle$ state. As a result, the photon energy required for LCP (σ+) and RCP (σ−) light absorption is different, resulting in CD signals in different wavelength regions. Recently, a theory was also proposed based on a chirality‐induced spin‐orbit coupling (SOC) to describe the natural optical activity of the chiral perovskites.^[^ ^44^ ^]^ Due to the presence of chirality‐induced SOC, the exciton dispersion has a parabolic structure with the energy minima occurring at a finite $K=\mp K_{0}=\mp \alpha \prime \tau M/\hbar^{2}$ for excitons with *J*_z_ = ±1, as shown in Figure 5a. $M=m_{e}+m_{h}$, in which *m*_e_ and *m*_h_ are electron's and hole's effective mass, respectively. *τ*(=±1) is material's helicity. $\alpha \prime \left(=\left(\alpha_{e}m_{e}+\alpha_{h}m_{h}\right)/M\right)$ is the exciton's SOC strength, in which *α*_e_ and *α*_h_ are electron's and hole's SOC strength, respectively. Clearly, $K_{0}$ changes sign when *J*_z_ and *τ* is reversed. The dispersions for excitons with *J*_z_ = ±1 are nondegenerate. For a given *K*_z_ = *q*, the absorption of LCP and RCP light generates excitons with *J*_z_ = 1 (red line) and *J*_z_ = −1 (blue line) at different energies, leading to the CD spectrum.

**Figure 4:**
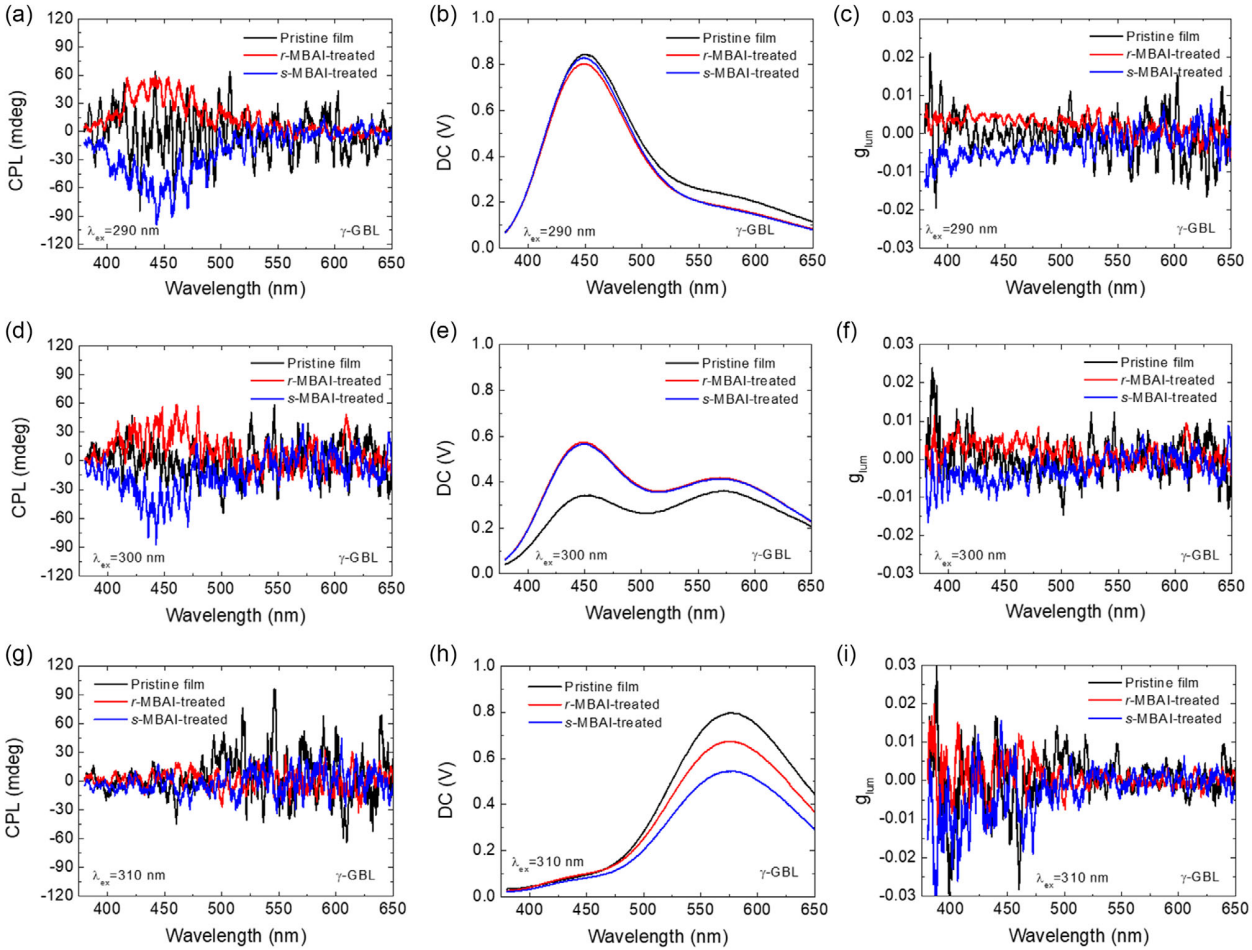
a,d,g) CPL spectra, b,e,h) DC voltages, and c,f,i) *g*_lum_ values of the pristine, *r*‐MBAI‐treated, and *s*‐MBAI‐treated halide perovskite films prepared from the precursor solution inγ‐GBL. (CsI:CuI = 1.15:2) The excitation wavelengths are set at (a),(b),(c) 290 nm, (d),(e),(f) 300 nm, and (g),(h),(i) 310 nm.

Room‐temperature CPL was also observed from the post‐treated lead‐free halide perovskite film prepared from the precursor with CsI to CuI molar ratio of 1.15:2. Unpolarized light of different wavelengths was used for excitation. The CPL spectra, the difference in emission intensity between the LCP and RCP light, were recorded at room temperature using a CPL spectrometer. The DC voltages, representing unpolarized PL intensity, and the luminescence dissymmetry factor $g_{\text{lum}}=\frac{0.000069913\times \text{ellipticity}\left(\text{mdeg}\right)}{\text{DC}\left(V\right)}$ were also recorded. Similar characteristics and trends of the lead‐free halide perovskite films prepared from different precursor solvents are observed and shown in **Figure** **4** and Figure S13, Supporting Information, respectively.

As shown in Figure 4b,e,h, S13b, S13e, and S13h, Supporting Information, the DC curves (PL spectra) change their peak wavelength with the excitation wavelength, which is similar to the excitation wavelength‐dependent PL spectra shown in Figure 2b and Figure S8b. When the excitation wavelength is 290 nm, the apparent CPL and *g*_hum_ value around ±0.005 were obtained around 445 nm, as shown in Figure 4a,c, S13a and S13c, Supporting Information. The CPL was observed at the same wavelength as the PL, which is attributed to the STEs.

**Figure 5:**
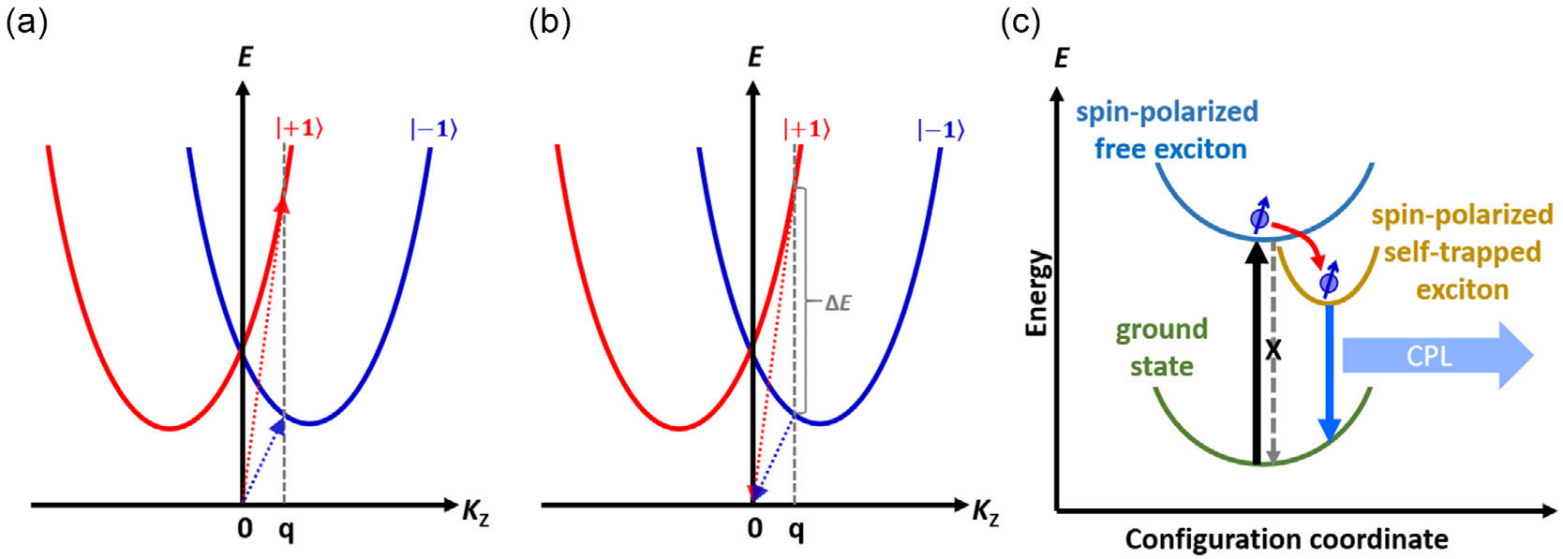
Schematic illustrations of the dispersion relation near *K*_z_ = 0 to explain a) CD and b) CPL. c) Schematic illustrations of the energy transfer from the spin‐polarized free exciton state to the spin‐polarized STE state.

As described previously, because of the presence of chirality‐induced SOC, the exciton's dispersions for *J*_z_ = ±1 are nondegenerate and form a double parabola. At finite temperatures, excitons gain kinetic energy with finite distribution at other *K* values and direct luminescence is possible once the exciton occupation at wavenumber $K=q=\sqrt{\varepsilon_{b}}\omega_{0}/c$ is appreciable (c is the speed of light, ℏ*ω*_0_ is the photon energy, and $\varepsilon_{b}$ is the dielectric constant excluding the exciton contribution).^[^ ^44^, ^45^ ^]^ For a given *K*_z_ = *q*, the intensities for LCP and RCP light are different because the LCP and RCP light originate from excitons in different bands with different exciton populations, as shown in **Figure** **5**b. In this work, the lead‐free chiral halide perovskite films exhibited broadband emission and large Stokes shifts attributed to the presence of STEs. CPL was also observed in the same PL wavelength region. Therefore, upon photoexcitation, the chiral halide perovskite first generates spin‐polarized free excitons with *J*_z_ = ±1, and then these excitons are transferred to the STE state. Since the energy transfer process is faster than the spin flip process,^[^ ^46^, ^47^, ^48^, ^49^ ^]^ the spin‐polarized free excitons can transfer to the STE state without losing their spin information through an energy and spin funneling process.^[^ ^47^, ^50^, ^51^ ^]^ The spin‐polarized STEs thus generate CPL, as shown in Figure 5c.

However, even when the excitation wavelength is 310 nm, the main DC curves (PL spectra) can be observed at 580 nm (Figure 4h and S13h, Supporting Information), but no significant CPL and *g*_lum_ value can be observed around 580 nm (Figure 4g,i, S13g and S13i, Supporting Information). In order to understand why CPL can only be observed around 445 nm, another set of perovskite films were prepared from the precursors with different molar ratios of CsI to CuI for CPL measurement again. As shown in Figure S14–S16, Supporting Information, CPL signals around 445 nm can be obtained from the Cs_3_Cu_2_I_5_ component. However, no apparent CPL signal can be obtained from CsCu_2_I_3_ around 580 nm.

All these CPL spectrum results indicate that CsCu_2_I_3_ does not produce CPL. Furthermore, it has been shown that there is no photogenerated carrier transfer between Cs_3_Cu_2_I_5_ and CsCu_2_I_3_. Based on the time‐resolved micro‐PL decay spectra of two different spots on the composite film, a previous report has shown that the Cs_3_Cu_2_I_5_ and CsCu_2_I_3_ grains in the composite film have similar lifetimes to the pure Cs_3_Cu_2_I_5_ and CsCu_2_I_3_ films, suggesting that there is no energy transfer between CsCu_2_I_3_ and Cs_3_Cu_2_I_5_ in the composites.^[^ ^12^ ^]^ Based on the temperature‐dependent PL of the composite film, with decreasing the temperature, the PL intensities of Cs_3_Cu_2_I_5_ and CsCu_2_I_3_ increase and the intensity ratio of them remains the same, suggesting the absence of photogenerated carrier transfer.^[^ ^12^ ^]^ If the situation is not as described above, the energy transfer takes place in the composite film, the excitons tend to transfer to the lower energy component and show mainly low energy PL peaks. Without energy transfer, two independent emission peaks can be observed synchronously under excitation to generate white light from the composite film. Therefore, based on our CPL experiment and the previous report,^[^ ^12^ ^]^ we believe that CsCu_2_I_3_ does not generate CPL and the spin‐polarized excitons do not transfer from Cs_3_Cu_2_I_5_ to CsCu_2_I_3_ to generate CPL.

## 3 Conclusion

In summary, the chiroptical activities of the chiral halide perovskite films composed of 0D Cs_3_Cu_2_I_5_ and 1D CsCu_2_I_3_ were investigated. Intrinsic chirality‐induced CD signals were observed at room temperature without the application of an external magnetic field, indicating the presence of chirality in the lead‐free chiral halide perovskites. Large Stokes shifts and broadband PL emission were observed, attributed to the presence of STEs. The CIE chromaticity coordinates (0.33, 0.33) were obtained. Room‐temperature CPL was also observed from the Cs_3_Cu_2_I_5_ component.

## 4 Experimental Section

4.1

4.1.1

##### Fabrication of the Perovskite Films

The precursor solutions were prepared in a mixture of DMF and DMSO (1:1 v/v) or inγ‐GBL. Three precursor solutions with CsI to CuI molar ratios of 2.75:2, 1.15:2, and 1.05:2 were prepared for spin coating. Toluene was used as the antisolvent during the spin coating process. After spin coating, the substrates were annealed at 100 °C for 30 min. The *r*‐MBAI (or *s*‐MBAI) was introduced on the perovskite films by dropping *r*‐MBAI (or *s*‐MBAI) toluene solution on top of the films, staying still for 30 s, spin drying the film, and annealing at 100 °C for 30 min.

##### Characterization

PL, PL excitation, and absorption spectra were recorded on an FS5 spectrofluorometer (Edinburgh Instruments). CD spectra were obtained on a JASCO J‐1500 spectrometer, whereas CPL spectra were recorded on a JASCO CPL‐300 spectrometer.

## Conflict of Interest

The authors declare no conflict of interest.

## Author Contributions

**Min‐Han Tsai**: investigation (lead); validation (lead). **Chia‐Hsiang Chuang**: investigation (supporting); validation (supporting). **Pei‐Hsuan Lo**: investigation (supporting); validation (supporting). **Wei‐Yun Zeng**: investigation (supporting); validation (supporting). **Chun‐Yao Huang**: investigation: (supporting); validation (supporting). **Lan‐Sheng Yang**: investigation (supporting); validation (supporting). **Yu‐Chiang Chao**: conceptualization (lead); writing—original draft (lead); writing—review editing (lead). **Min‐Han Tsai**, **Chia‐Hsiang Chuang** and **Pei‐Hsuan Lo** contributed equally to this work.

## Supporting information

Supplementary Material

## Data Availability

The data that support the findings of this study are available from the corresponding author upon reasonable request.
