# Supplementary material for: Chiroptical Activities of Low‐Dimensional Lead‐Free Chiral Halide Perovskites with White‐Light Emission
Source: Small Sci. 2025 May 15;5(6):2500034. doi: 10.1002/smsc.202500034 (PMC12168611; doi:10.1002/smsc.202500034)
Supplement: Supplementary file 1 — Supplementary Material [file SMSC-5-2500034-s001.pdf]

Supporting Information

Chiroptical Activities of Low-Dimensional Lead-Free Chiral Halide Perovskites with White-Light Emission

*Min-Han Tsai, Chia-Hsiang Chuang, Pei-Hsuan Lo, Wei-Yun Zeng, Chun-Yao Huang, Lan-Sheng Yang, and Yu-Chiang Chao**

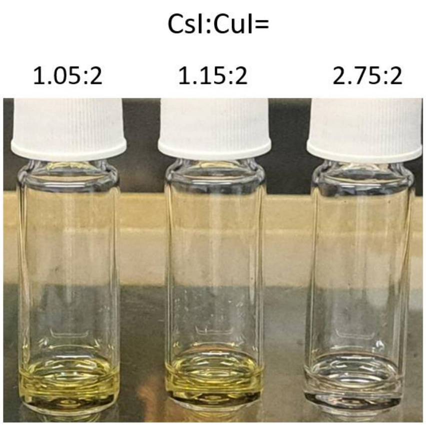

Figure S1. The photograph shows the precursor solutions with CsI to CuI molar ratios of 2.75:2, 1.15:2, and 1.05:2.

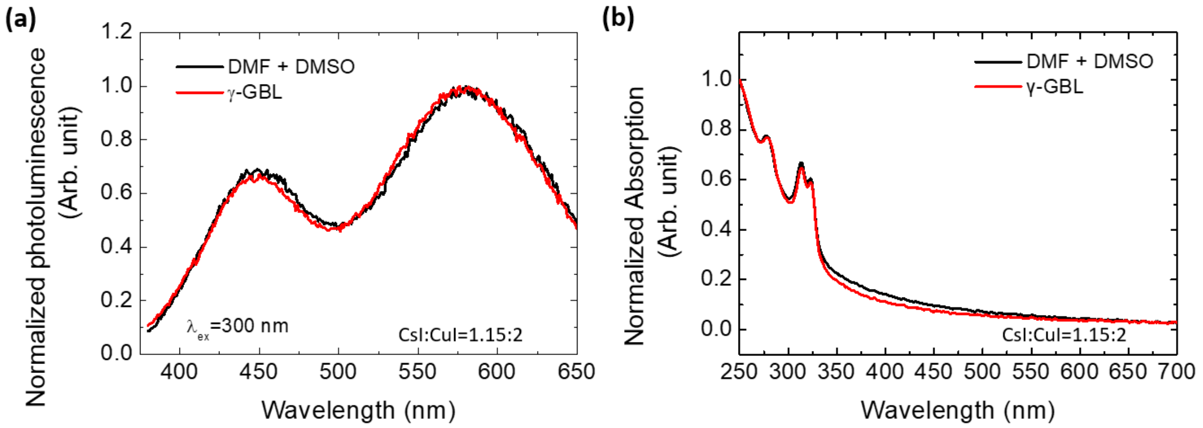

Figure S2. (a) Photoluminescence and (b) absorption spectra of the perovskite films prepared from precursor solutions in different solvents.

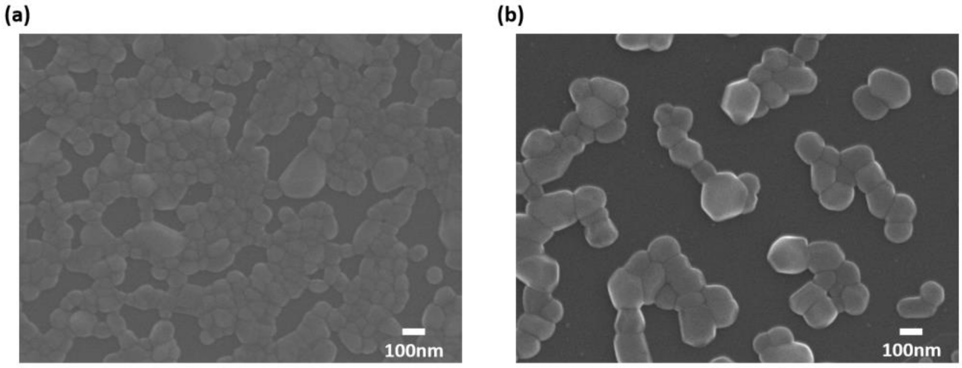

Figure S3. SEM images of the perovskite films (a) before and (b) after treatment with *r*-MBAI toluene solution.

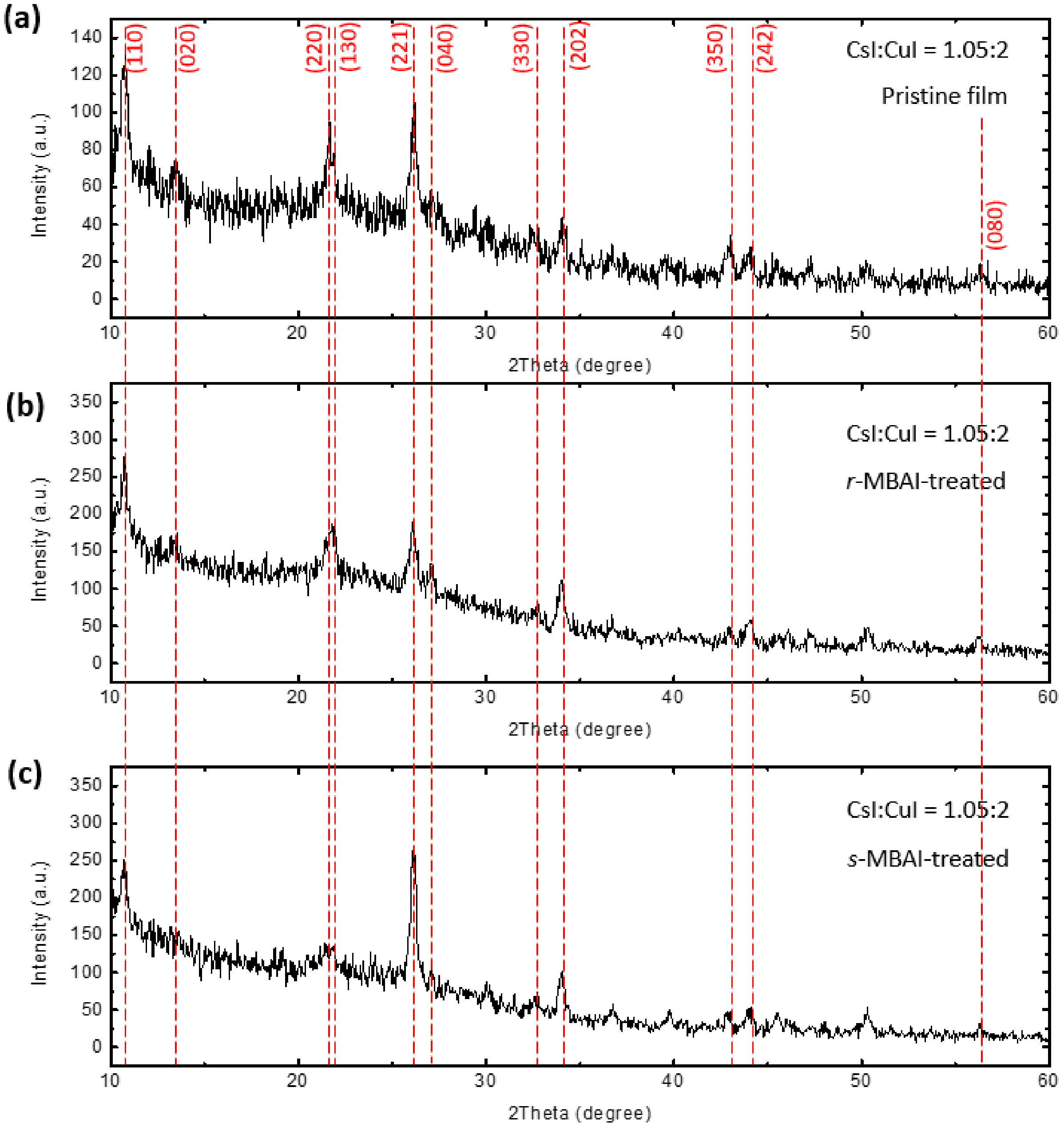

Figure S4. The XRD pattern of the (a) pristine, (b) *r*-MBAI-treated, and (c) *s*-MBAI-treated perovskite films prepared from the precursor with a CsI to CuI molar ratio of 1.05:2.

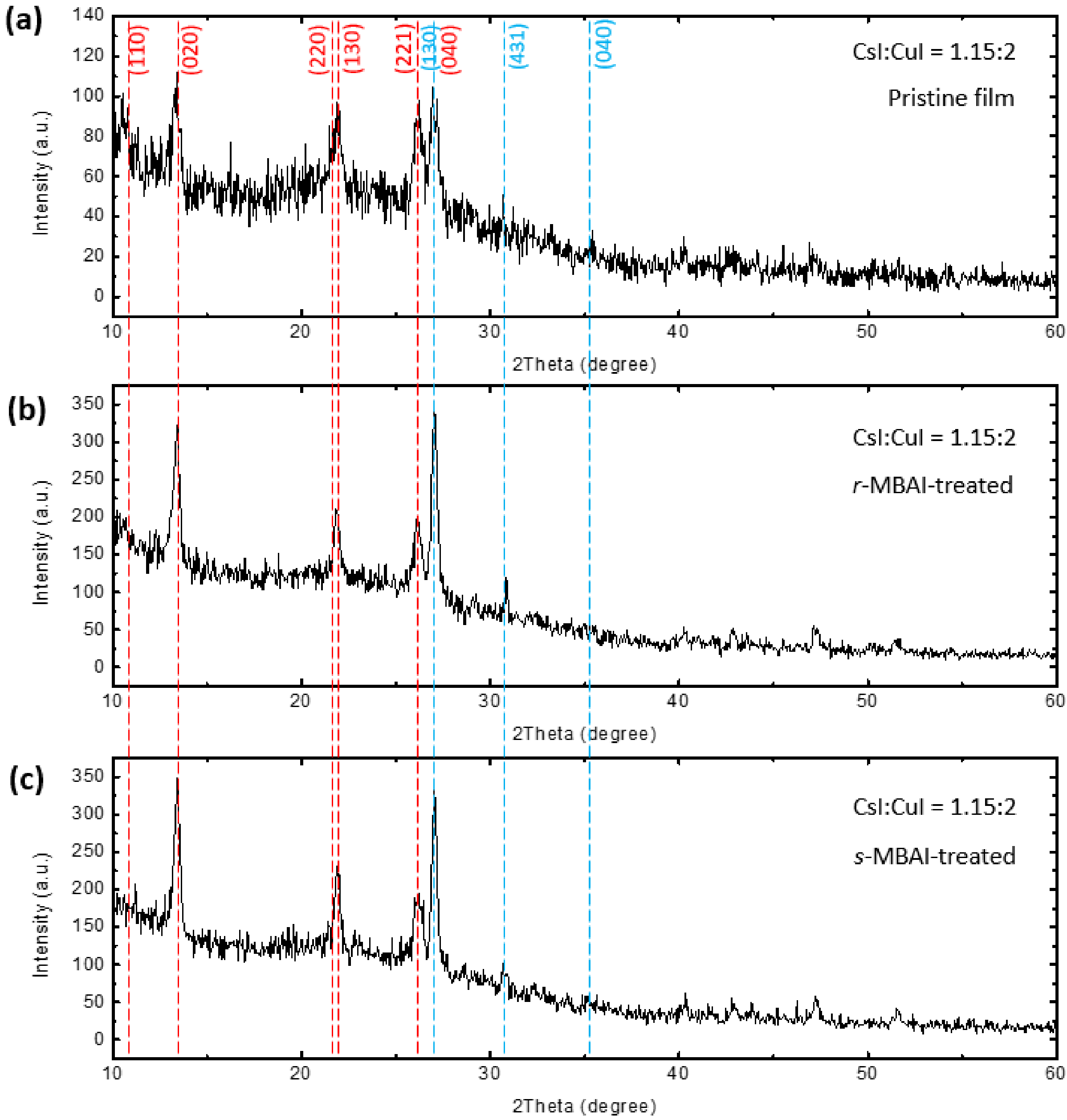

Figure S5. The XRD pattern of the (a) pristine, (b) *r*-MBAI-treated, and (c) *s*-MBAI-treated perovskite films prepared from the precursor with a CsI to CuI molar ratio of 1.15:2.

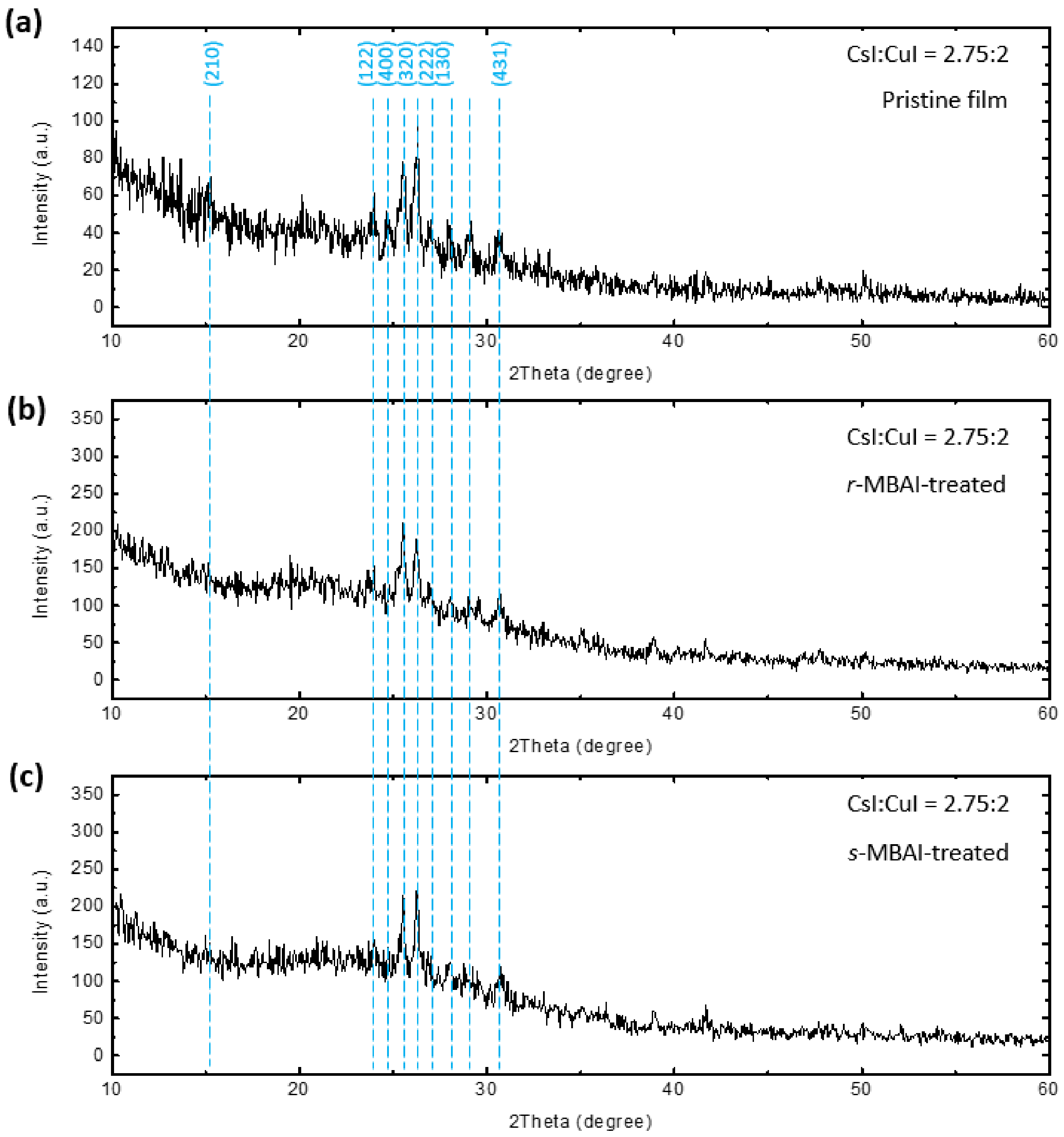

Figure S6. The XRD pattern of the (a) pristine, (b) *r*-MBAI-treated, and (c) *s*-MBAI-treated perovskite films prepared from the precursor with a CsI to CuI molar ratio of 2.75:2.

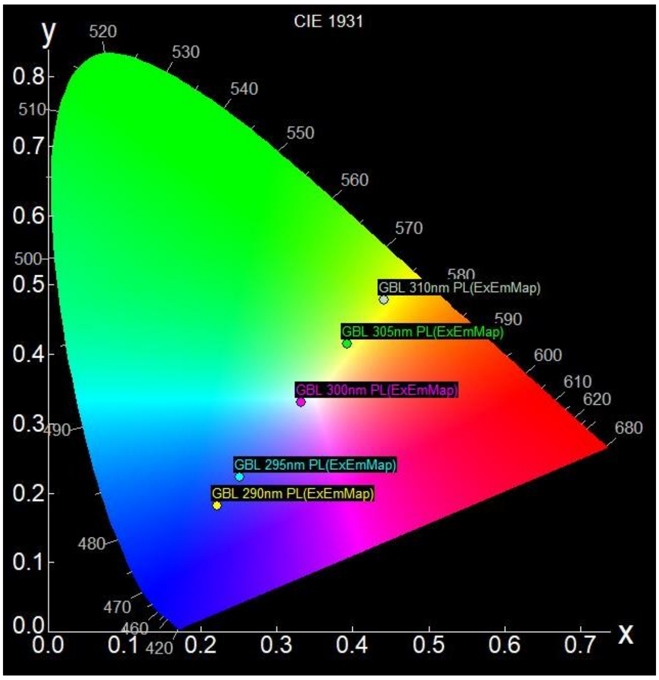

Figure S7. The CIE chromaticity coordinates under different excitation wavelengths of the perovskite films prepared from γ-GBL.

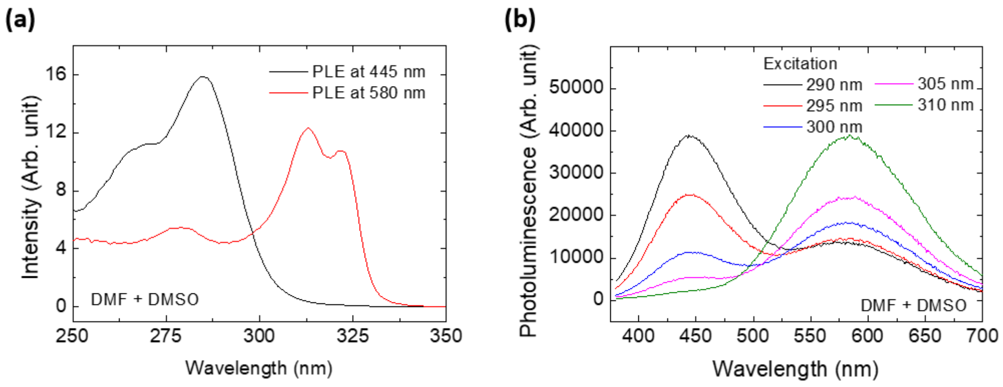

Figure S8. (a) Photoluminescence excitation spectra measured at 445 nm and 580 nm. (b) Photoluminescence spectra measured at different excitation wavelengths. The solvent used to prepare the precursor solution is DMF+DMSO.

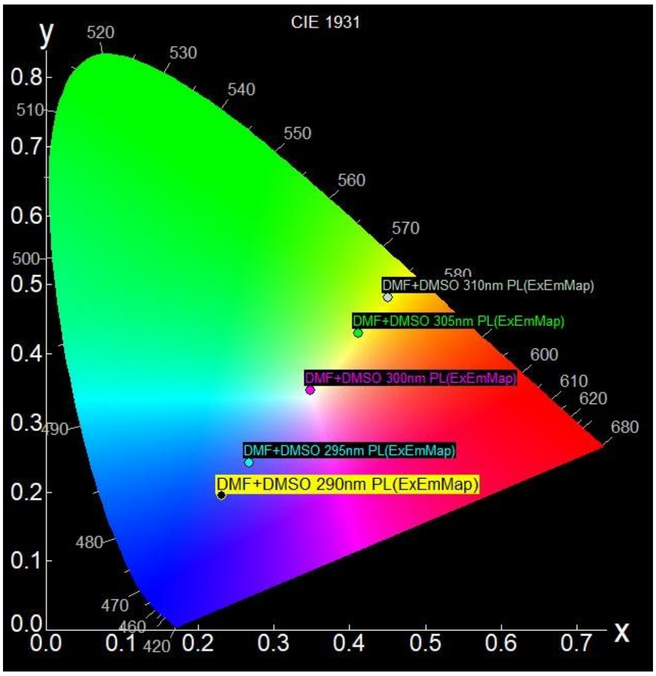

Figure S9. The CIE chromaticity coordinates under different excitation wavelengths of the perovskite films prepared from DMF/DMSO.

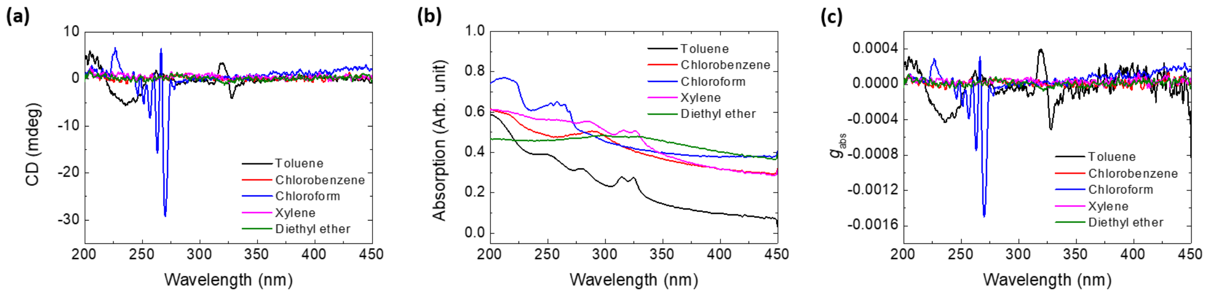

Figure S10. (a) CD spectra, (b) absorption spectra, and (c) g_{abs} values of the perovskite films post-treated with *r*-MBAI in different solvents.

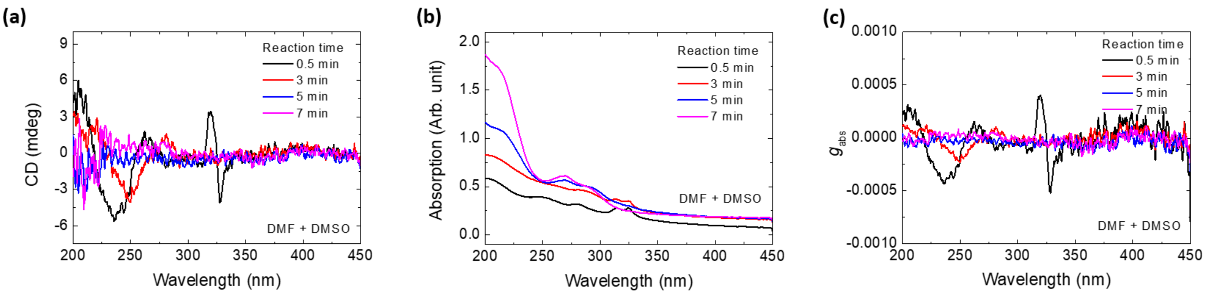

Figure S11. (a) CD spectra, (b) absorption spectra, and (c) g_{abs} values of the perovskite films post-treated with *r*-MBAI toluene solution for different times.

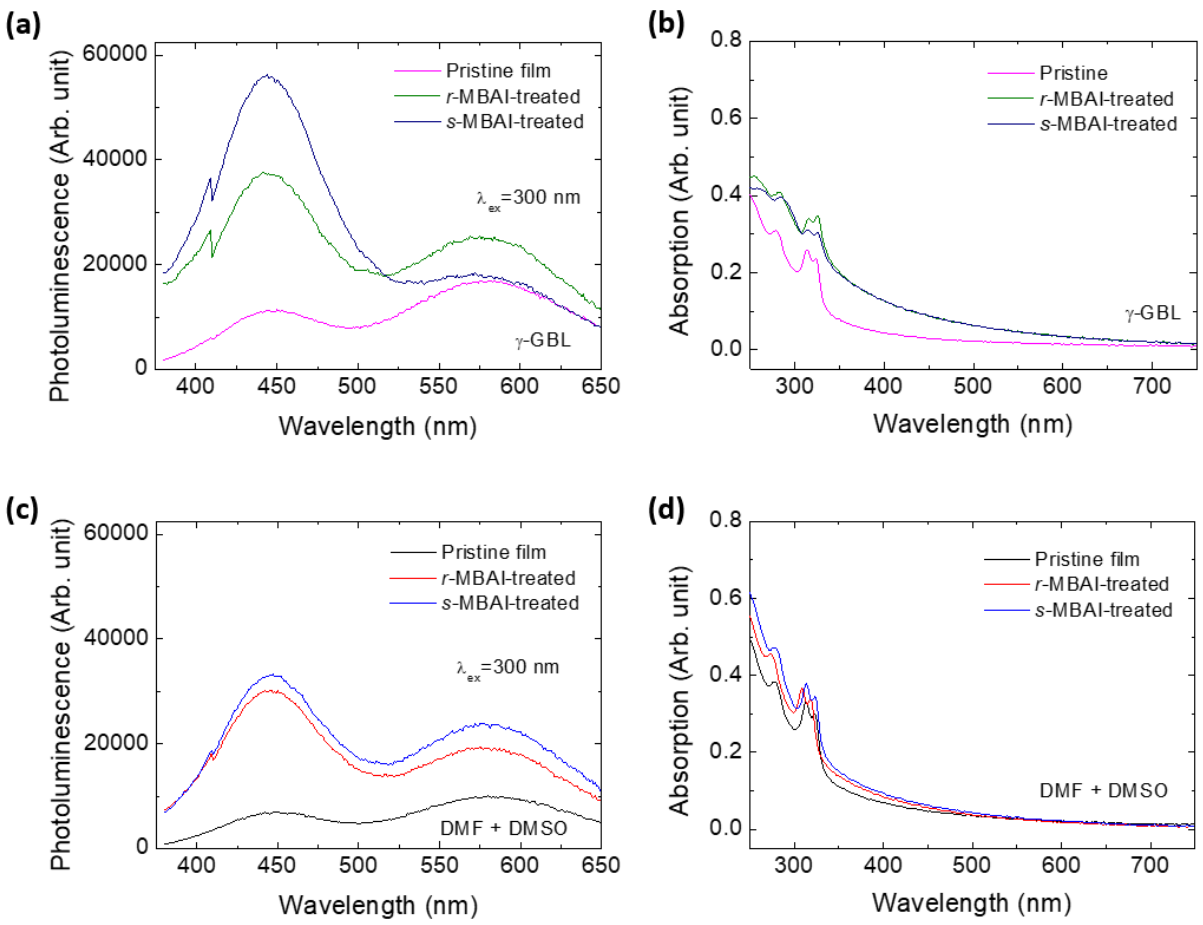

Figure S12. (a),(c) PL spectra, and (b),(d) absorption spectra of the pristine, *r*-MBAI-treated, and *s*-MBAI-treated perovskite films prepared from the precursor solution in (a),(b) γ-GBL and (c),(d) DMF/DMSO.

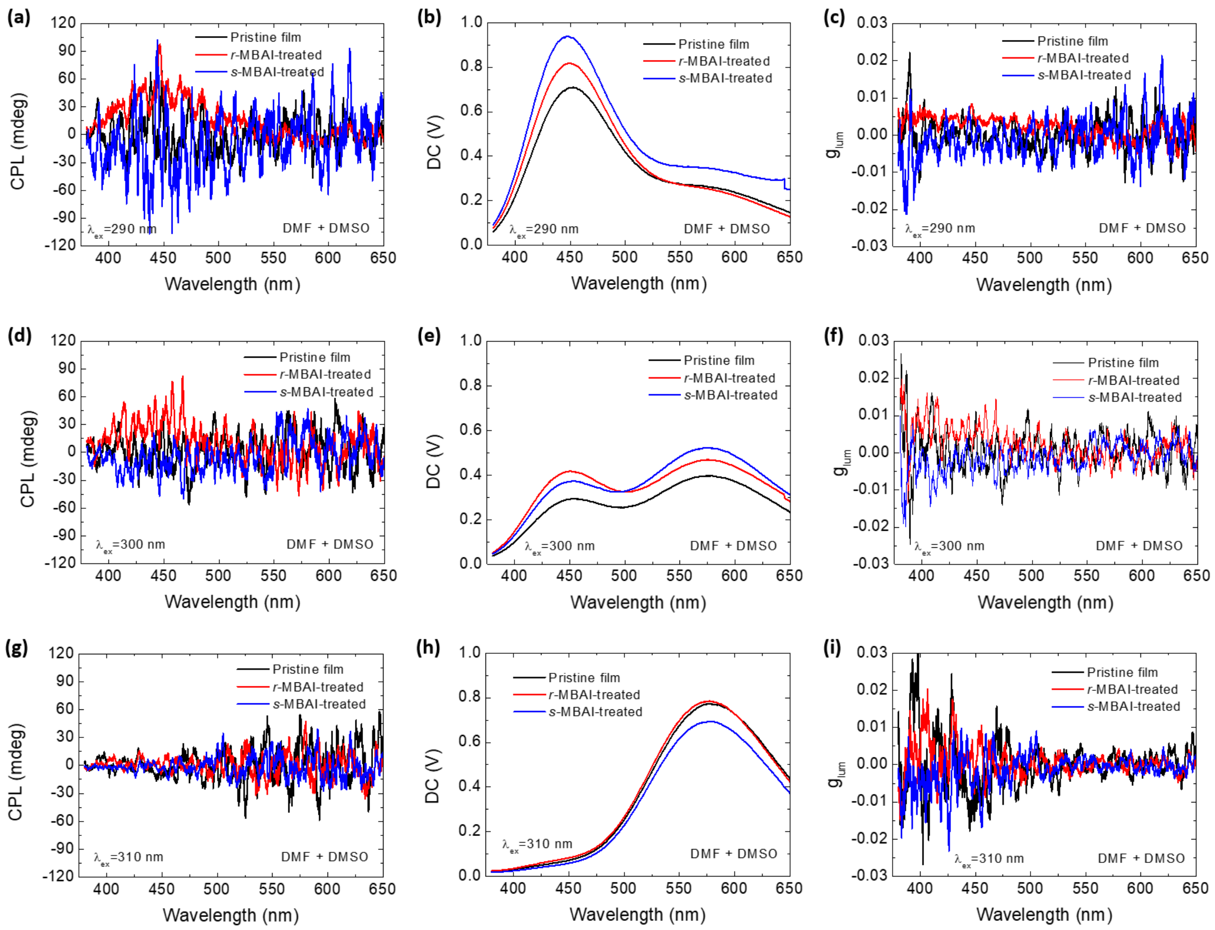

Figure S13. (a),(d),(g) CPL spectra, (b),(e),(h) DC voltages, and (c),(f),(i) g_{lum} values of the pristine, *r*-MBAI-treated, and *s*-MBAI-treated halide perovskite films prepared from precursor solution in DMF/DMSO. (CsI:CuI=1.15:2) The excitation wavelengths are set at (a),(b),(c) 290 nm, (d),(e),(f) 300 nm, and (g),(h),(i) 310 nm.

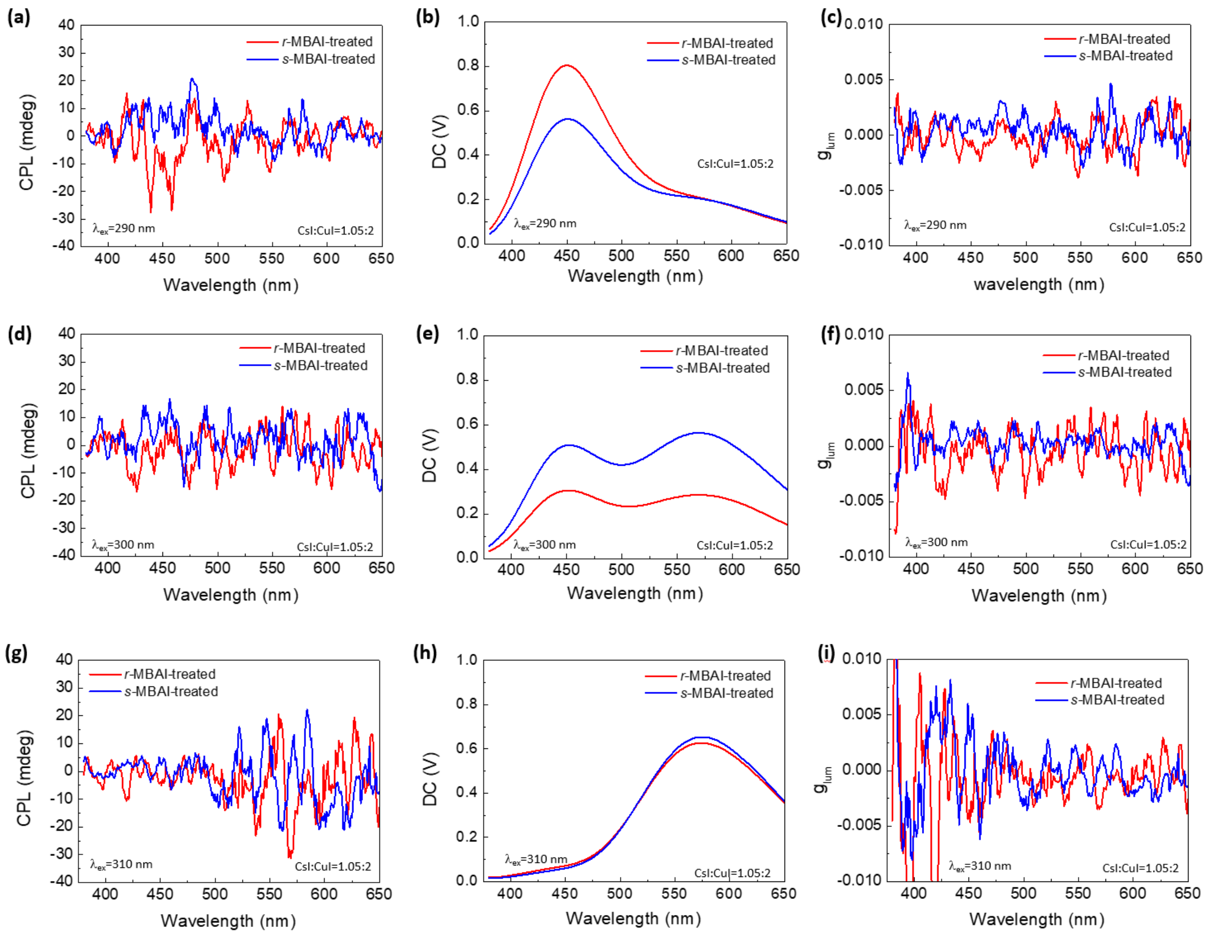

Figure S14. (a),(d),(g) CPL spectra, (b),(e),(h) DC voltages, and (c),(f),(i) g_{lum} values of the pristine, *r*-MBAI-treated, and *s*-MBAI-treated halide perovskite films prepared from precursor solution in γ-GBL. (CsI:CuI=1.05:2) The excitation wavelengths are set at (a),(b),(c) 290 nm, (d),(e),(f) 300 nm, and (g),(h),(i) 310 nm.

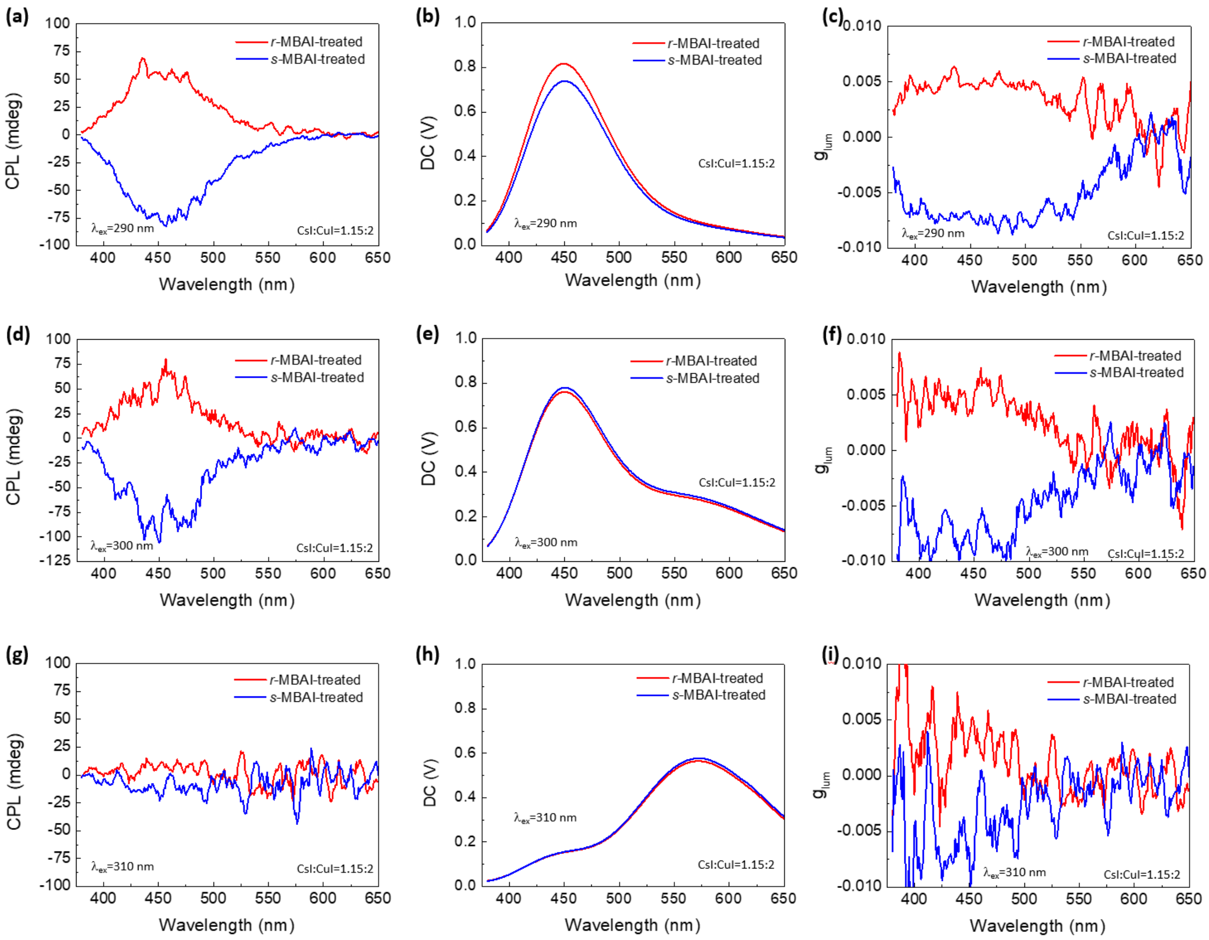

Figure S15. (a),(d),(g) CPL spectra, (b),(e),(h) DC voltages, and (c),(f),(i) g_{lum} values of the pristine, *r*-MBAI-treated, and *s*-MBAI-treated halide perovskite films prepared from precursor solution in γ-GBL. (CsI:CuI=1.15:2) The excitation wavelengths are set at (a),(b),(c) 290 nm, (d),(e),(f) 300 nm, and (g),(h),(i) 310 nm.

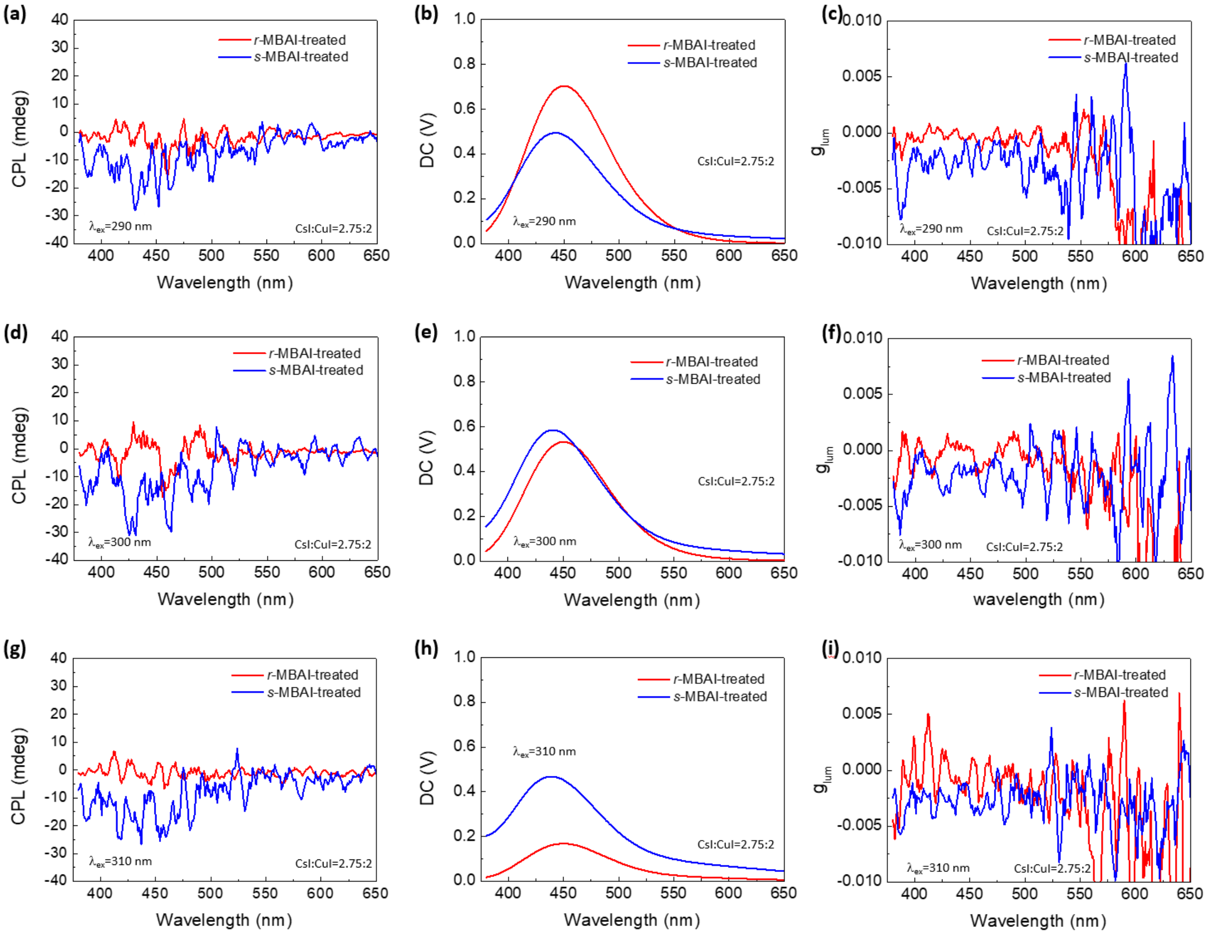

Figure S16. (a),(d),(g) CPL spectra, (b),(e),(h) DC voltages, and (c),(f),(i) g_{lum} values of the pristine, *r*-MBAI-treated, and *s*-MBAI-treated halide perovskite films prepared from precursor solution in γ-GBL. (CsI:CuI=2.75:2) The excitation wavelengths are set at (a),(b),(c) 290 nm, (d),(e),(f) 300 nm, and (g),(h),(i) 310 nm.
